# Chronobiotics KL001 and KS15 Extend Lifespan and Modify Circadian Rhythms of *Drosophila melanogaster*

**DOI:** 10.3390/clockssleep3030030

**Published:** 2021-08-20

**Authors:** Ilya A. Solovev, Mikhail V. Shaposhnikov, Alexey A. Moskalev

**Affiliations:** 1 Laboratory of Geroprotective and Radioprotective Technologies, Institute of Biology, Komi Science Centre, Ural Branch, Russian Academy of Sciences, Kommunisticheskaya Str. 28, 167982 Syktyvkar, Russia; ilyasolovev-ksc@yandex.ru (I.A.S.); mshaposhnikov@mail.ru (M.V.S.); 2 Medical Institute, Pitirim Sorokin Syktyvkar State University, Oktyabrsky Prosp. 55, 167001 Syktyvkar, Russia

**Keywords:** chronobiotics, geroprotectors, cryptochrome, KL001, KS15, *Drosophila melanogaster*

## Abstract

Chronobiotics are a group of drugs, which are utilized to modify circadian rhythms targeting clock-associated molecular mechanisms. The circadian clock is known as a controller of numerous processes in connection with aging. Hypothesis: KL001 and KS15 targeting CRY, affect lifespan, locomotor activity and circadian rhythm of *Drosophila melanogaster*. We observed a slight (2%, *p* < 0.001) geroprotective effect on median lifespan (5 µM solution of KL001 in 0.1% DMSO) and a 14% increase in maximum lifespan in the same group. KS15 10 µM solution extended males’ median lifespan by 8% (*p* < 0.05). The statistically significant positive effects of KL001 and KS15 on lifespan were not observed in female flies. KL001 5 µM solution improved locomotor activity in young male imagoes (*p* < 0.05), elevated morning activity peak in aged imagoes and modified robustness of their circadian rhythms, leaving the period intact. KS15 10 µM solution decreased the locomotor activity in constant darkness and minimized the number of rhythmic flies. KL001 5 µM solution improved by 9% the mean starvation resistance in male flies (*p* < 0.01), while median resistance was elevated by 50% (*p* < 0.0001). This phenomenon may suggest the presence of the mechanism associated with improvement of fat body glucose depos’ utilization in starvation conditions which is activated by dCRY binding KL001.

## 1. Introduction

Natural selection determined the formation of diverse molecular systems responsible for reception and transduction of light signals as time in the biosphere as well as in society has the central role. The molecular timer is called the circadian (circa—near, dian—day) clock or molecular oscillator [1]. The clock is characterized by the robustness of generated rhythms [2]. The periodicity found in behavioural and biochemical patterns and in gene expression profiles is synchronized with the rhythms of Earth’s rotation by zeitgebers [3]. Zeitgeber is a dominating stimulus which resets the clock. There are different zeitgebers like light, temperature, food intake, physical activity and numerous endogenous metabolites (Heyde, I.; Oster, 2021) [4]. Below we sought to describe the oscillator of *Drosophila melanogaster* which is the model used in our study.

The CLK/CYC dimer, whose elements are encoded by the *Clock* (*Clk*) and *cycle* (*cyc* or *dBmal1*) genes, forms a positive feedback loop by interacting with the “E-box” enhancer sequences in the *period* (*per*) and *timeless* (*tim*) gene promoters and induces their expression at the dusk. TIM and PER proteins accumulate in the cell nucleus late at night and interact with the CLK/CYC dimer. As a result, CLK/CYC is inactivated; the result of this interaction is the suppression of *per* and *tim* transcription until the moment when the PER/TIM repressor dimer does not degrade, and the positive feedback does not prevail over the negative one. That is, until the oscillator restarts [5]. Diurnal fluctuations are further enhanced through post-translational modifications of the oscillator proteins, affecting the conformation of its domains, especially through sequential phosphorylation [6]. Although fluctuations persist under photo deprivation conditions, synchronization usually occurs relative to photoregimen via the *cry* gene-encoded photoreceptor flavoprotein CRY. Flavin adenine dinucleotide acts as a chromophore molecule in it. When a quantum of light hits the CRY molecule, the latter binds to the TIM protein, inducing its degradation [7]. The TIM protein stabilizes PER; the latter, following CRY, and degrades with the participation of the DBT factor several hours after activation [6]. The differences between mammal and insect clock are minor, but significant. TIM is not involved in the mammalian repressor heterodimer; this protein rather acts as a regulator of the cell division cycle. A vacant place in the protein complex is occupied by the flavoproteins CRY1 or CRY2. CYC in mammals is absent; its ortholog is BMAL1. In mammalian cells, there is a protein that doubles the function of *Drosophila*’s CLK, its symbol is NPAS2 [6]. Another significant difference between invertebrate circadian oscillators is the multiplicity of genes for the determinants of circadian rhythms; this feature is even more pronounced in plants. Two or more paralogs increase the stability of the system, so it is possible to disorganize circadian rhythms by induction of mutations in each related sequence or by interferential inactivation of all gene variants [8].

Oscillators’ correct functioning is essential for the optimal growth, predators’ avoidance and protection from exogenous challenges like oxidative damage, irradiation and temperature elevation. There is evidence that the proper timekeeping is responsible for healthy aging and is associated with longevity [9,10]; the aging itself may affect circadian rhythmicity [11,12,13]. Data obtained on model animals and humans showed that an impaired circadian clock weakens the response of the organism to exo- and endogenous stressors; exogenous stressors are environmental challenges mainly and endogenous are considered to be aging, associated oxidative stress (damage of macromolecules by oxidation products), loss of proteostasis, decompartmentalization, loss of biological barriers, etc. Circadian rhythm of xenobiotics’ detoxification affects the pharmacokinetics and dynamics of the drugs which was utilized as a basal principle of chronotherapy when patients are administered to strictly rhythmic doses of drugs. Nowadays the paradigm changed in search of the appropriate time of binding of the target, which expression is under the circadian control, this principle is also called circadian medicine [14].

Chronotherapy as a working model for geroprotective interventions is built on current evidence that: (1) circadian rhythms ebb away during aging [15,16,17], (2) metabolic disorders may be induced by circadian disruption [18,19,20] which leads to lifespan decrease [21,22,23,24], while health may be restored together with circadian rhythmicity [14,25] and longevity [22,23] and (3) aging-associated pathways may oscillate within a circadian period [26,27].

The improvement of circadian oscillations of gene expression is associated with upregulation of the oscillator’s elements (such as *per*) and which was properly shown in *Drosophila* [2,28]. Earlier we have shown that ectopic conditional overexpression in different tissues prolongs lifespan of *Drosophila melanogaster* and improves flies’ stress-resistance [28]. Studying the age-related changes in gene expression in the total *Drosophila* homogenate, we observed the gradual deficit of *cry* transcripts [28]. This phenomenon may be explained in the framework of the hypothesis of age-related photoresistance [29]. The biological sense of photoresistance is in the lack of light-receptor molecules observed in old *Drosophila*. The compensation and supercompensation of *cry* deficit extend the lifespan as it improves the adaptivity of the fly to the light stimuli and enhances the response to oxidative damage (constant light conditions are known as a factor accelerating the aging process) [29].

Small molecule modulators of circadian rhythms, also known as chronobiotics, can be subdivided into several classes which are formed according to tendencies to bind a direct target included in circadian oscillator molecular mechanism: casein kinase (CKs) interactors, cryptochrome (CRYs) ligands, RORs interactors and REV-ERBs ligands and specific GSK-3 inhibitors [30,31]. Casein kinases inhibitors (for CK I) are presented by Longdaysin, DK359, NCC007, PF4800567, Epiblastins A and C, for CK II we may report about DMAT, TBB, CX-4945, and GO289 [30]. GSK-3-specific inhibitors are known as CHIR99021, BRD1652 [30]. RORs interactors (mainly agonists) are presented by T0901317, SR1001, SR2211, SR1555, SR3335, SR1078, Nobiletin, and Neuroruscogenin [30]. The group of REV-ERB ligands includes compounds GSK4112, SR9009, SR10067, GSK2945, SR8278, and ARN5187 [30]. The subgroup of CRY ligands is known for cryptochrome activators KL001, Compound 50, KL044, GO200, KL101, TH301, etc. and inhibitors KS15-derivatives and non ethoxipropanoic derivatives [31,32].

We may also define a type of indirect clock modulators; these compounds tend to affect the expression profile of clock genes and modify periodicity. Among indirect clock modulators it is possible to sort out phototransduction modifiers (opsinamides are melanopsin inhibitors), non-specific GSK-3 inhibitors (Lithium, Benzodiazepine derivatives), *Bmal1*-inducers (L-methyl selenocysteine), *Sirt1*-activators (Resveratrol) and chronobiotics with unknown targets (Compound 10/CEM3) [33,34]. The geroprotective potential of the ccompounds mentioned above is poorly studied, except resveratrol and lithium.

We chose the dCRY protein as a target since the genetic interventions in its expression do show high anti-aging potential, but pharmacological interventions have not yet been described in the literature. We hypothesize that pharmacological stabilization of dCRY extends the lifespan of *D. melanogaster*. To check our hypothesis, we chose specific ligands of cryptochrome flavoprotein: activator KL001 and inhibitor KS15 [33]. The mentioned drugs are known as cryptochrome specific modulators of circadian rhythms in cell cultures, and noteworthy is the great number of clock modulators that have already been discovered.

KL001 and KS15 were selected as the compounds of interest for the study because earlier we had found the impact of dCRY in *Drosophila* aging and longevity [28,29]. We needed both activator and inhibitor of dCRY to shed light on mechanisms of geroprotection. 

The goal of this study is in visualizing organismal circadian rhythms of locomotory activity under the influence of two chronobiotics utilized in doses shown as effective ones in case of life extension.

## 2. Methods

Climatic chambers Binder KBF720-ICH (Binder, Germany) were used for keeping fruit flies. The flies were kept at a temperature of 25 °C, a relative humidity of 60% and a 12-h illumination regime.

The wild-type strain *Canton-S* of *Drosophila melanogaster* was used as a model animal (#64349, Bloomington, IN, USA). The number of dead flies was counted daily. The insects were transplanted onto a fresh medium twice a week. The composition of the medium per 1 L of water: 7 g—agar-agar, 8 g—dry yeast, 30 g—granulated sugar, 30 g—semolina, 8 mL—both 50% propionic acid (as a fungicide) and 10% solution of nipagin in 96% ethanol.

To study the effects of the cryptochrome activator, 30 μL of a KL001 solution (Sigma-Aldrich, St. Louis, MO, USA) in 0.1% DMSO water solution at concentrations of 1, 5, 10, and 50 μM were applied to the surface of the *Drosophila* culture medium. On the medium of the control group of *D. melanogaster*, 30 μL of 0.1% DMSO was applied.

To study the effects of a cryptochrome inhibitor KS15 (GlixxLabs, Hopkinton, MA, USA) 30 μL of a KS15 solution in distilled water and 0.1% DMSO at concentrations of 1, 5, and 10 μM were applied to the surface of the *Drosophila* culture medium. On the medium of the control group of flies, 30 μL of 0.1% DMSO was applied. Water was used as a negative control of all experiments where the longevity of the flies was measured.

The concentration of 50 μM has not been studied; the cytotoxic effects of KS15 in high concentrations have been previously described [35].

Data for locomotory activity and circadian rhythms measurement were obtained with the use of DAMSystem (TriKinetics, USA). The output files were uploaded to web-based software “ShinyR-DAM v3.1 “Refresh”” [36], to analyze and visualize locomotor activity, sleep parameters and circadian rhythms parameters. The flies were kept in glass capillaries with agarose medium (2% agar, 5% sucrose) and the surface was covered with yeast paste containing drugs and 8 μL of 10% solution of nipagin in 96% ethanol to prevent fungal growth during the experiment. The period of activity examination lasted six days, and precise measurements were taken of pure LD (12:12) and DD periods, two and four days, subsequently. The tests of locomotor activity were carried out only on male flies to avoid the signal aberration due to oviposition and larval locomotion in the narrow glass tube. The age of flies was five days after imago eclosion at the date when they were placed on synchronisation, and the measurements were made for 6–11th days and 34–41st days of imago’s life.

The test of starvation resistance was made on 2% agar-agar medium with 8 mL of 50% propionic acid per litre and a solution of nipagin in ethanol to prevent fungal growth during the experiment.

Demographic methods were used to study the effects of KL001 on the lifespan of the *Drosophila melanogaster* strain of the wild type, *Canton-S*. Statistical analysis was carried out in software Statistica 6, Microsoft Excel and in web application OASIS 2 [37].

*Statistics.* Mean lifespan distributions were compared by log-rank test in stress-resistance tests [37]. Medians and mortality percentiles were compared with Gehan-Breslow-Wilcoxon and Wang-Allison tests [28,38].

Median mortality differences in stress resistance were estimated by an exact Fisher’s test [39]. To estimate the differences between circadian rhythmicity and locomotion of groups receiving different treatment we used ANOVA with Tukey/Kramer and Scheffe’s multiple comparison procedures along with Mann-Whitney tests [40] All the statistical tests were chosen according to the experience of the earlier published papers in the field [28,29].

## 3. Results and Discussion

*Effects of KL001 on Drosophila melanogaster lifespan.* Statistically significant results of the assessment of differences in indicators in groups receiving activator KL001 at different concentrations indicate the ability of KL001 to prolong the lifespan of Drosophila. Therefore, for a concentration of 5 μM, an increase of 3.5% is shown for the average lifespan, *p* < 0.00001, according to the χ^2^-test), 2% for median lifespan (50% of population mortality), *p* < 0.001, in accordance to the Gehan-Breslow-Wilcoxon test (Table 1). The age of 90% mortality of the population that received 5μM KL001 with food increased by 14%, *p* < 0.05, is in accordance to the Wang-Allison test. The time of 90% mortality of the population was subjected to a statistically significant effect of KL001 in all variants of the experiment; there was an increase in the indicator by 9–14%, *p* < 0.05, according to the Wang-Allison test.

An experiment to establish the effect of the KL001 cryptochrome activator on lifespan was also carried out on females of the Canton-S strain. In an experiment on *Drosophila melanogaster* females, a weak effect of the KL001 on lifespan parameters was revealed. The only statistically significant effect was an 18% increase in median lifespan in the case of the group that received KL001 as a solution with a concentration of 1 μM in 0.1% DMSO, *p* < 0.05 according to the Wang-Allison test. In the experiment, where a concentration of KL001 of 5 μM was used on females there was a tendency to an increase in the median lifespan by 18% at *p* = 0.0534; however, it is not a statistically significant result. Significant results in females receiving KL001 were verified by (*p* < 0.05) Wang-Allison test, with a 36% increase in median lifespan of 5 µM KL001 cohort and 33% increase in median of 50 µM KL001 group, relatively to DMSO control; however, the statistically significant results lack in maximal lifespan studies.

*Effects of KS15 on Drosophila melanogaster lifespan.* There is an increase in the average lifespan of males receiving KS15 at a concentration of 10 μM (*p* < 0.05). An increase in median lifespan by 8% (*p* < 0.05) was also recorded (according to the Gehan-Breslow-Wilcoxon test). The results of evaluation of the differences between the samples, calculated using the Wang-Allison test indicated no statistically significant positive effects.

The analysis of the survival of females treated with KS15 in different concentrations with food, in accordance with the results of statistical analysis, revealed tendencies towards prolongation of median lifespan when obtaining a concentration of 1 µM and 10 µM.

The graphical representation of all survival-testing experiments is given in the Supplementary Figure S1 and raw data which was used to draw the survival curves is presented in Supplementary Table S1.

We observed the principal differences in DMSO sensitivity in male and female flies. According to our data, DMSO was of high toxicity for *Canton-S* female flies, while the males’ lifespan parameters were not affected by the solvent (Table 2). We observed also the same slight, but statistically significant effect of 5 µM KL001 solution in males, and even in relative to negative control the flies showed minor life extension. We did not observe the effects on maximal lifespan comparing the experimental group with the negative control in males.

Comparing the male and female experimental and control groups with negative control (water-treated) we observed the same tendencies for DMSO, a decrease in the lifespan of female individuals and no influence to the male lifespan parameters in the majority of cases. We did not observe the statistical differences between water and DMSO groups’ medians in males (*p* = 0.0661, Gehan-Breslow-Wilcoxon test), while the median lifespan in female control relative to negative control was 41% smaller (*p* < 0.00001, Gehan-Breslow-Wilcoxon test). The same situation is characterized in the result obtained for control groups of females treated with DMSO solutions of KS15 (Table 3).

**Figure 1:**
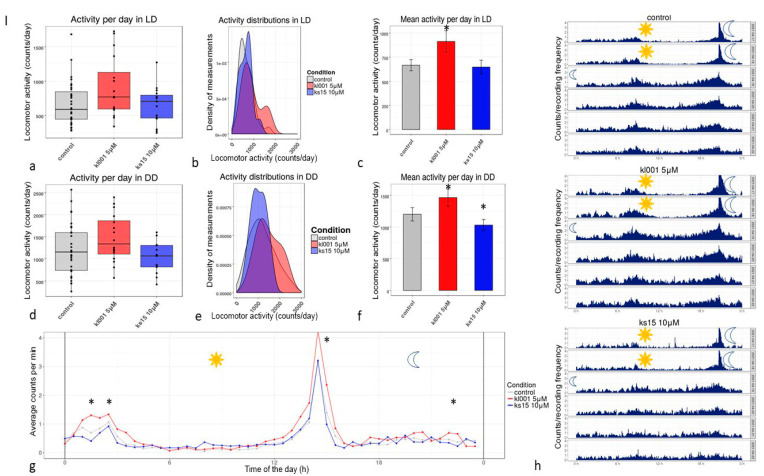
The locomotor activity profiles measured for CS males treated with lifespan modifying doses of KL001 и KS15 until the age of 5–12 days: (**a**,**d**)—activity per day in LD and DD; (**b**,**e**)—activity distributions in LD and DD; (**c**,**f**)—mean activities in LD and DD; (**g**)—profile of locomotor activity in LD, measured as an average per minute; (**h**)—actograms, presenting and comparing all the profile during six days of test, first to lines are LD, last four—DD in all three blocks; *—*p* < 0.0001 significant results, according to ANOVA with Tukey/Kramer procedure, *p* < 0.01 with Scheffe’s procedure for KS15 (**c**,**f**); *—*p* < 0.05, Mann-Whitney test for control vs. KL001 comparison. The effect on locomotor activity was observed both in LD and DD for KL001 cohort and for KS15 in DD, the daily activity profile in LD was elevated by KL001 5 µM solution in young age.

*Effects on locomotor activity, sleep and circadian rhythms.* The chronobiotics KL001 and KS15 were studied as modulators of organismal circadian rhythms in concentrations (5 µM and 10 µM, subsequently) which were detected as effective geroprotective ones. We tested only male flies as chronobiotics’ treatment resulted in life extension only in this group, and additional explanation is in avoidance of larval movement. The results observed in cohorts treated with KL001 or KS15 indicate the vivid chronotherapeutic potential of both pharmacological agents and locomotor activity modulatory effect in KL001. The daily locomotor activity value in average representation was 37% higher (*p* < 0.0001, ANOVA with Tukey Kramer procedure, *p* < 0.001 with Scheffe’s procedure) in LD (12 h:12 h) regimen and 22% higher in DD (free run, constant darkness) for KL001 (*p* < 0.00001, ANOVA with Tukey Kramer procedure, *p* < 0.0001, also *p* < 0.001 with Scheffe’s procedure) treated cohort (Figure 1c,f). Average activity profiles for KL001 treated flies (Figure 1g) differ much, especially at the peak regions (late night and early evening elevations) (*p* < 0.0001, ANOVA with Tukey/Kramer procedure, *p* < 0.001 with Scheffe’s procedure).

**Figure 2:**
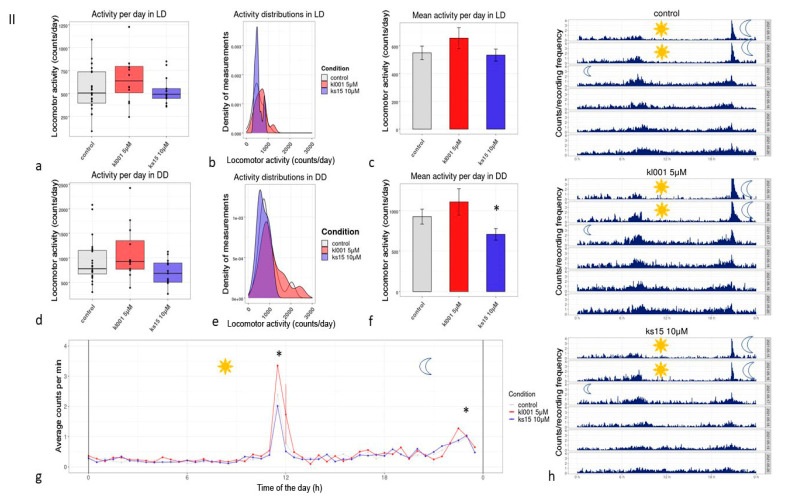
The locomotor activity profiles measured for CS males treated with lifespan modifying doses of KL001 и KS15 until the age of 34–41 days: (**a**,**d**)—activity per day in LD and DD; (**b**,**e**)—activity distributions in LD and DD; (**c**,**f**)—mean activities in LD and DD; (**g**)—profile of locomotor activity in LD, measured as an average per minute; (**h**)—actogramms, presenting and comparing all the profile during six days of test, first to lines are LD, last four—DD in all three blocks; *—*p* < 0.0001 significant results, according to ANOVA with Tukey/Kramer procedure, *p* < 0.001 with Scheffe’s procedure (**c**,**f**); *—*p* < 0.05, Mann-Whitney test for control vs. KL001 comparison (**g**). In older age we observed only the effect on locomotor activity in a group which received 10 µM solution of KS15.

At the age of 34–41 days, flies treated with KL001 do not show any significant changes in locomotor activity patterns; the age-related alteration in activity peak coincides with the control group treated with DMSO (Figure 2). The activity peaks at the later age were still higher in the KL001 group (Figure 2g) *p* < 0.05, with the Mann-Whitney test for control vs. KL001 for comparison.

KS15 significantly reduced mean locomotor activity in the later age (Figure 2f), with a *p* < 0.0001 significant results, according to ANOVA with Tukey/Kramer procedure, and *p* < 0.01 with Scheffe’s procedure. KS15 did not affect the activity profile significantly.

**Figure 3:**
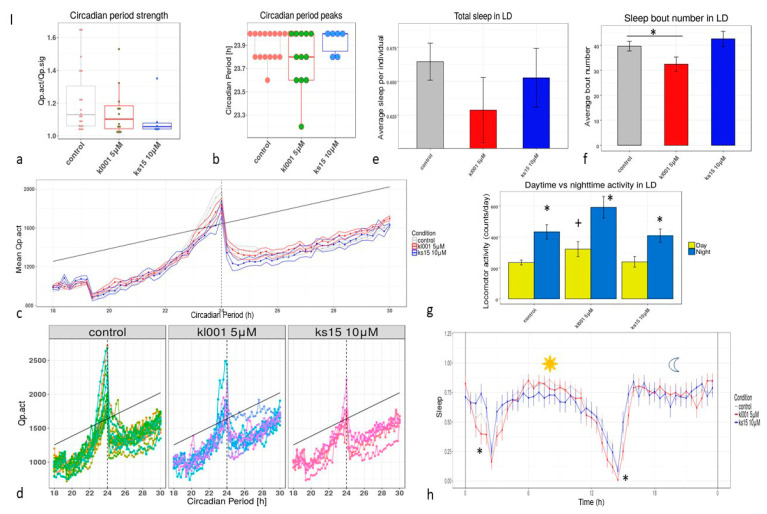
The parameters of male flies’ circadian rhythms, sleep/activity profile until the age of 5–12 days: (**a**)—circadian period robustness; (**b**)—circadian period peaks’ quantitative representation; (**c**)—mean periodogram, (**d**)—individual periodograms, (**e**)—total sleep in LD, (**f**)—sleep bout number in LD, *—*p* < 0.05, Mann-Whitney test; (**g**)—comparative diagram daytime vs. night-time activity in LD, *—0.001 ANOVA with Tukey/Kramer procedure, also *p* < 0.01 with Scheffe’s procedure, for light and dark periods of the day, +—*p* < 0.001—for light and light in different cohorts by treatment, (**h**)—sleep profile the differences in local distributions were measured with ANOVA for the control and KL001 groups. The chronobiotics did not significantly alter the period of the circadian rhythms in male flies of young age, but seriously affected the sleep profile in KL001-treated cohort; the sleep bout number decreased by 20% (*p* < 0.05). The total activity was higher in daytime in the KL001 cohort as well as in night-time. Night-time activity was higher in all cohorts relative to daytime but not in the control group.

**Figure 4:**
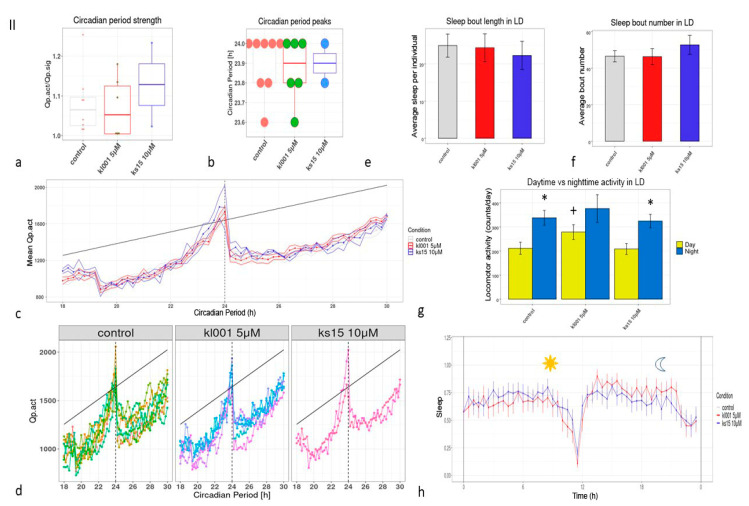
The parameters of male flies’ circadian rhythms, sleep/activity profile until the age of 34–41 days: (**a**)—circadian period robustness; (**b**)—circadian period peaks’ quantitative representation; (**c**)—mean periodogram, (**d**)—individual periodograms, (**e**)—total sleep in LD, (**f**)—sleep bout number in LD, *—*p* < 0.05, Mann-Whitney test; (**g**)—comparative diagram daytime vs. nighttime activity in LD, *—*p* < 0.001 ANOVA with Tukey/Kramer procedure, also *p* < 0.01 with Scheffe’s procedure, for light and dark periods of the day, +—for light and light in different cohorts by treatment, (**h**)—sleep profile for the differences in local distributions were measured with ANOVA for the control and KL001 groups. We observed only a total daytime activity elevation in the KL001 cohort of old age (*p* < 0.01).

Studying the sleep parameters of the KL001 group we verified the elevation of activity during the day and night registering less sleep bouts than in the control group treated with DMSO (Figure 3h). Both daytime and night-time activities were higher in males treated with KL001 (*p* < 0.05, Mann-Whitney test) (Figure 3g). Circadian activity measured for DD conditions was significantly altered by both pharmacological agents; KL001 slightly reduced the robustness of circadian rhythms when KS15 disorganized the rhythms (Figure 3a). We observed an insignificant decrease (Mann-Whitney test, *p* > 0.05) in the median period of oscillations of the locomotor activity value (Figure 3b), with 24-h peaks in individual periodograms dominating in all three samples. On the other hand, KL001 in mammals is described as a compound which elongates the circadian period due to accumulation of CRY [41]. In the later age of imagoes (34–40 days), the tested flies did not show any unique circadian effects which are associated with administration to both drugs. The only statistically significant effects were in the test of day and night activity; activity during the day was higher in the group which was treated during all life with 5 µM solution of KL001 (Figure 4g).

We see the sense of this phenomenon in organizational differences in molecular clock mechanisms; in insects, CRY is a receptor and the transductor of the zeitgeber stimulus and is not working directly as a repressor. Considering the hypothesis of age-related photoresistance [29], the effect of lifespan extension may be explained as a result of CRY accumulation (the overexpression of *cry* in different tissues may increase lifespan in *Drosophila*) [29,42].

Numerous CRY-activators like KL001 are well known for their positive effects on metabolism, especially this group of compounds which affects gluconeogenesis taking place in hepatocytes. It is possible that the impact on lifespan and locomotor activity increase is associated with the phenomenon of more effective gluconeogenesis suppression by stabilized dCRY [41,43].

The effect of *cry^01^* mutation on starvation resistance was discussed earlier; the ability to survive was decreased on a 2%-agar medium compared with *w^1118^* which was used as a genetic background [29]. To prove the existence of a possible mechanism improving fat body glucose depos’ utilization in starvation conditions, we carried out an experiment testing the males’ resistance to the abovementioned environmental challenge using the dose of KL001 extending lifespan (5 µM).

The unsophisticated method indicates 9% (*p* = 0.0041) improvement in mean and 50% (*p* = 0.000042) increase in median starvation resistance after three days of treatment by 5 µM KL001 in male flies. We tend to think that modulation of glucagon-like pathways by cryptochrome is a very evolutionary conservative mechanism in animals. In our experiments it may explain the observed effects of KL001 on median and maximal lifespans [44,45,46]. It is known that in *Drosophila* the glucagon-like pathway controls both lipid and carbohydrate homeostasis, which are critical for lifespan determination [44,45,46].

Interestingly, KL001 may be a narcological drug: in male mice it induces a blockade of alcohol excessive and “relapse” drinking, activating CRY1. This finding has a high value for improvement of safe pharmacological behavioural correction methods used in alcohol abuse treatment to prolongate patients’ lifespan and improve quality of life [47].

Drug KS15 is better known to have anti-cancer traits than metabolic-modulatory ones [35]. KS15 reduces the pace of cancer cell proliferation and elevates the effectiveness of tamoxifen and doxorubicin in MCF-7 cells [35]. The accumulation of CRY1 in mammal’s cancer cells can stop PTX-induced (model senescence) by induction of p53 degradation. This mechanism possibly exists in *Drosophila* but is appropriate only for explanation of stem cell senescence rate decrease in imago, because the fruit fly is majorly a postmitotic organism.

The toxic effect of DMSO was earlier shown in the paper by Cvetković et al. (2015) [48]. The influence of solvent was detected especially in females, and this phenomenon may be associated with the amounts of the compound which were eaten by the experimental groups of females as the decrease in lifespan is observed in all variants [48].

## 4. Conclusions

The chronobiotics KL001 and KS15 can modulate organismal circadian rhythms in *Drosophila melanogaster*. Both drugs can extend the lifespan of male flies. KL001 increases locomotor activity after five days of treatment. Additionally, the 5 µM KL001 solution may improve starvation response and reduce the number of sleep bouts in males. Neither KL001 nor KS15 affect circadian period length but significantly reduce the rhythm’s robustness in young imagoes.

It is worth noting that several mechanisms of CRY-mediated life extension may exist. One of them is associated with stabilization of CRY protein and relative increase in its presence in the cell. Second mechanism is based on metabolic effects of CRY targeting. The inhibitory case (also leads to accumulation of CRY in the cell) is alternative and relates to an anti-senescence effect found in cancer cell culture, and focused on p53 degradation, may be also present in a fruit fly [26].

## Figures and Tables

**Table 1:** Effects of KL001 on *Drosophila melanogaster* males’ survival under starvation conditions.

Treatment	N	Survival Time (h)			Percentiles of Mortality (h)			
		Mean	±SEM	95% C.I.	25%	50%	75%	90%
0.1% DMSO (control)	118	38.44	0.87	36.74~40.15	32	32	48	48
0.1% DMSO, 5 µM KL001	125	41.82 *	1	39.87~43.78	32	48 ^+^	48	60

Note: χ^2^ = 8.25, *—*p* = 0.0041; +—*p* < 0.0001 (*p* = 0.000042, precise Fischer’s test).

**Table 2:** The effects of KL001 on *Drosophila melanogaster* lifespan.

Treatment	Sex	N	Mortality Percentiles (Days)			
			25%	50%	75%	90%
H_2_O (negative control)	♂	181	48	58	62	68
0.1% DMSO (control)	♂	152	54	58	58	60
0.1% DMSO, 1 µM KL001	♂	152	53	57	60	63 +
0.1% DMSO, 5 µM KL001	♂	151	52	59 *#@	63	66 +
0.1% DMSO, 10 µM KL001	♂	155	54	54	58	65 +
0.1% DMSO, 50 µM KL001	♂	148	54	58	61	64 +
H_2_O (negative control)	*♀*	134	58	72	75	79
0.1% DMSO (control)	*♀*	152	30	33	54	64
0.1% DMSO, 1 µM KL001	*♀*	149	29	39 +	57	64
0.1% DMSO, 5 µM KL001	*♀*	151	28	45 +	56	63
0.1% DMSO, 10 µM KL001	*♀*	152	26	37	51	58
0.1% DMSO, 50 µM KL001	*♀*	153	30	44 +	52	61

*—*p* < 0.001 according to the Gehan-Breslow-Wilcoxon test; +—*p* < 0.05 (0.0413) according to the Wang-Allison test, #—*p* = 0.0268, comparison with negative control (water-treated group), Gehan-Breslow-Wilcoxon test; @—*p* < 0.0001, Wang Allison test compared with water-treated cohort; the underlined values were significantly statistically different from negative water-treated control, the 4–7th columns had *p* < 0.0001, Wang-Allison test, the 5th column was tested with Gehan-Breslow-Wilcoxon test and all values had *p* < 0.0001); the *p*-values are mentioned with Bonferroni correction. The most prominent geroprotective effects on lifespan were observed in male flies. N—Number of flies.

**Table 3:** The effects of KS15 on *Drosophila melanogaster* lifespan.

Treatment	Sex	N	Mortality Percentiles (Days)			
			25%	50%	75%	90%
H_2_O (negative control)	♂	181	48	58	62	68
0.1% DMSO (control)	♂	145	52	59	64	67
0.1% DMSO, 1 µM KS15	♂	142	53	58	64	64
0.1% DMSO, 5 µM KS15	♂	153	50	57	64	64
0.1% DMSO, 10 µM KS15	♂	148	56 +	64 * **#^b^	64	72
H_2_O (negative control)	*♀*	134	58	72	75	79
0.1% DMSO (control)	♀	151	28	42	49	56
0.1% DMSO, 1 µM KS15	♀	148	28	42	53	60
0.1% DMSO, 5 µM KS15	♀	161	29	42	45 +	50
0.1% DMSO, 10 µM KS15	♀	155	35	43	49	64

*—*p* < 0.01, according to χ^2^-test; **—*p* < 0.01 according to the Gehan-Breslow-Wilcoxon test; +—*p* < 0.05 (*p* = 0.0432) according to the Wang-Allison test, #—*p* < 0.01, Wang Allison test compared with water-treated cohort; ^b^ — *p* < 0.0001, Wang Allison test compared with water-treated cohort, the underlined values were significantly statistically different from negative water-treated control, the 4–7th columns had *p* < 0.0001, Wang-Allison test, the 5th column was tested with Gehan-Breslow-Wilcoxon test and all values had *p* < 0.0001) all the *p*-values were measured with Bonferroni correction. We observed only a geroprotective effect of 10 µM concentration in male flies. N—Number of flies.

## Data Availability

MDPI Research Data Policies.

## Supplementary Materials

The following are available online at https://www.mdpi.com/article/10.3390/clockssleep3030030/s1, Figure S1: Survival curves of *Drosophila melanogaster* cohorts treated with chronobiotics KL001 and KS15 with two controls, Table S1: Raw survival data of flies treated with KL001 and KS15.
