# Supplementary material for: Chronobiotics KL001 and KS15 Extend Lifespan and Modify Circadian Rhythms of Drosophila melanogaster
Source: Clocks Sleep. 2021 Aug 20;3(3):429–41. doi: 10.3390/clockssleep3030030 (PMC8395451; doi:10.3390/clockssleep3030030)
Supplement: Supplementary file 1 [file clockssleep-03-00030-s001.zip › Supplementary file 1.pdf]

Supplementary file 1

Survival curves of Drosophila melanogaster cohorts treated with chronobiotics KL001 and KS15 with two controls

Note: the figures show in each case the curves for negative and positive controls and only one cohort of treated flies for convenience and comprehensibility of a reader, all the figures are drawn according to raw data files from Supplementary 2.

Chronobiotics KL001 and KS15 extend lifespan and modify circadian rhythms of *Drosophila melanogaster*

I. A. Solovev[1,2], M. V. Shaposhnikov[1], A. Moskalev[1]

[1]Laboratory of Geroprotective and Radioprotective Technologies, Institute of Biology, Komi Science Centre, Ural Branch, Russian Academy of Sciences, Kommunisticheskaya St. 28, Syktyvkar, 167982, Russian Federation;

[2]Medical Institute of Pitirim Sorokin Syktyvkar State University, Oktyabrsky prosp. 55, Syktyvkar, 167001, Russian Federation

Corresponding author: A.A. Moskalev (amoskalev@ib.komisc.ru)

Supplementary figure S1 KL001-treated males' survival

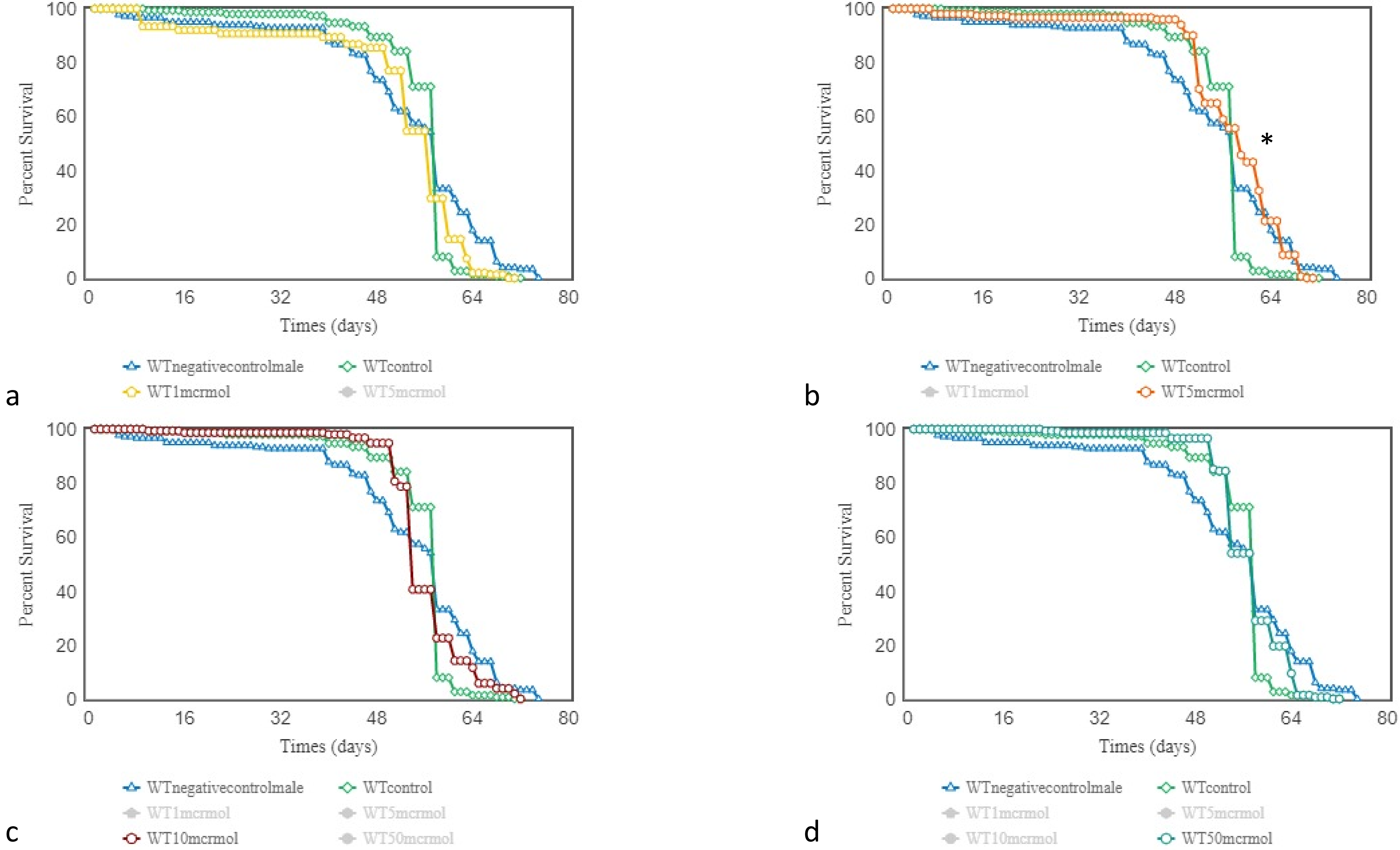

*- p= 0.000012, according to Log-Rank Test with Bonferroni correction (comparison with DMSO control)

Supplementary figure S2 KL001-treated females' survival

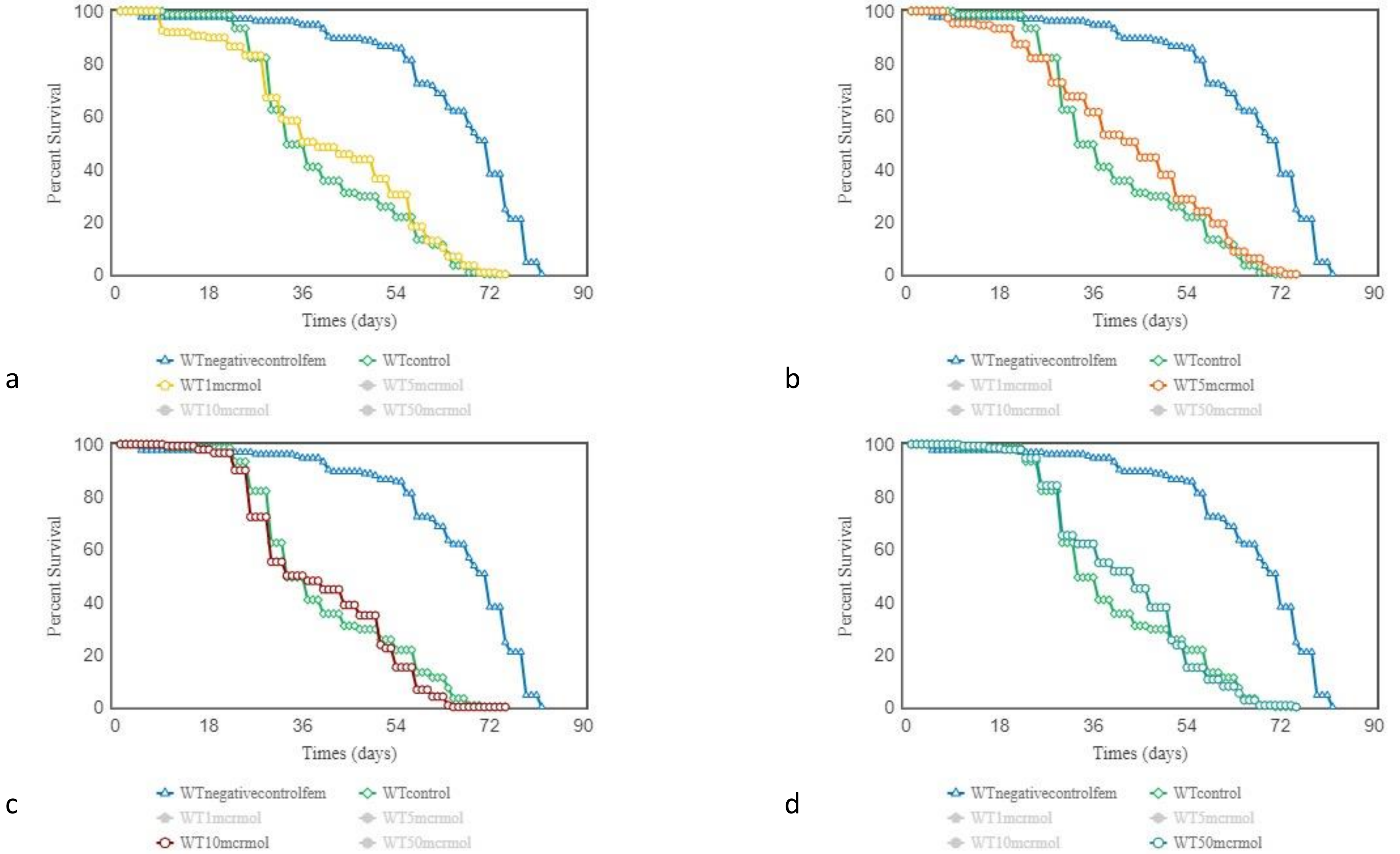

Supplementary figure S3 KS15-treated males' survival

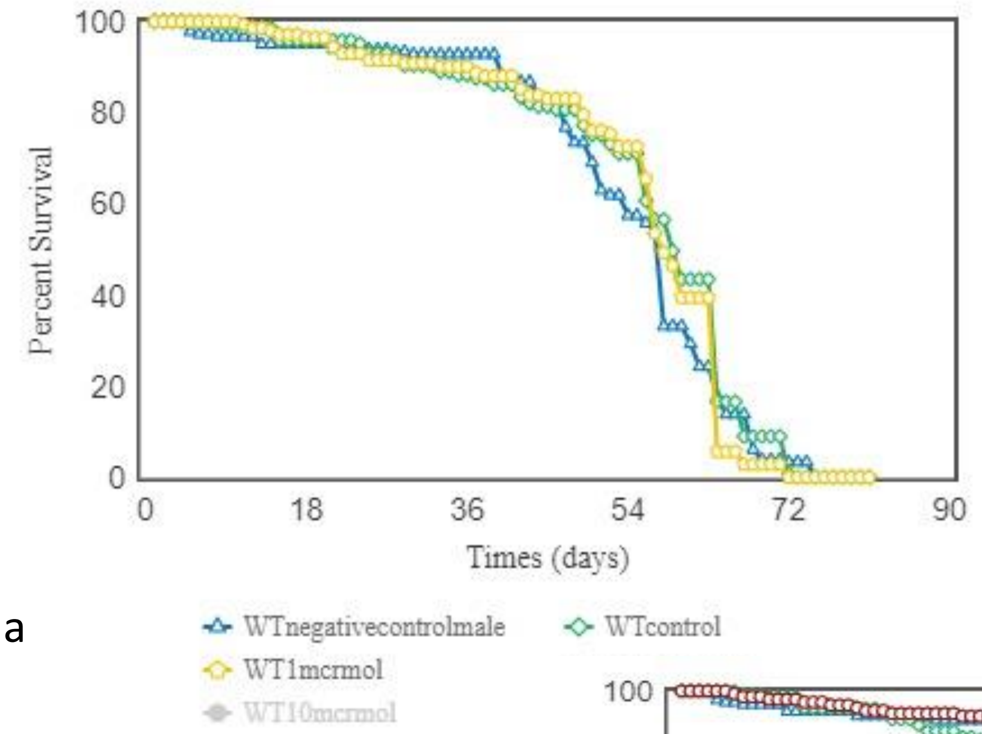

a

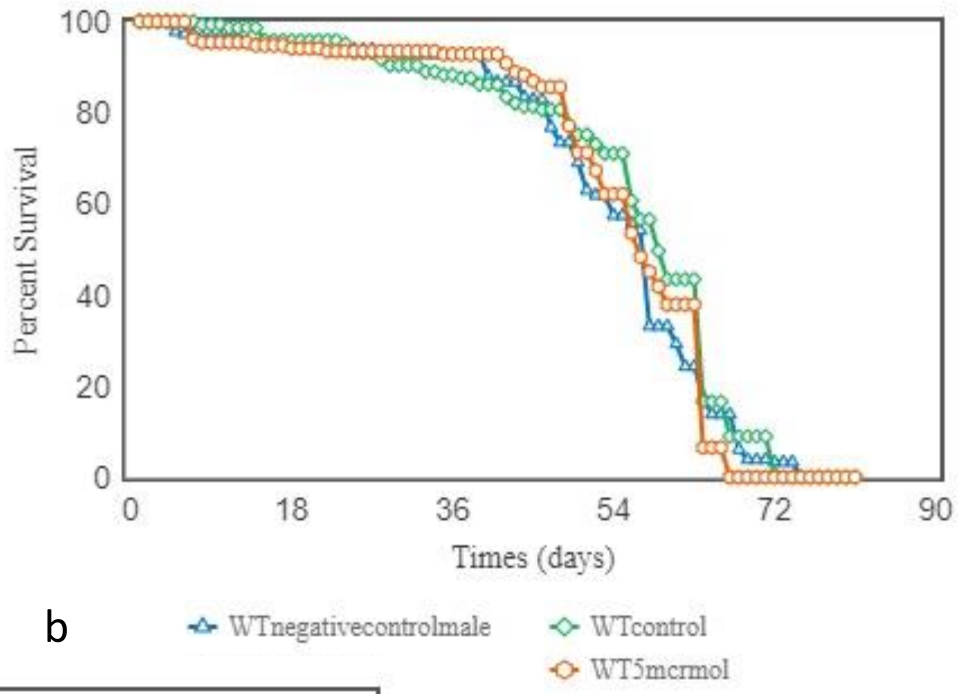

b

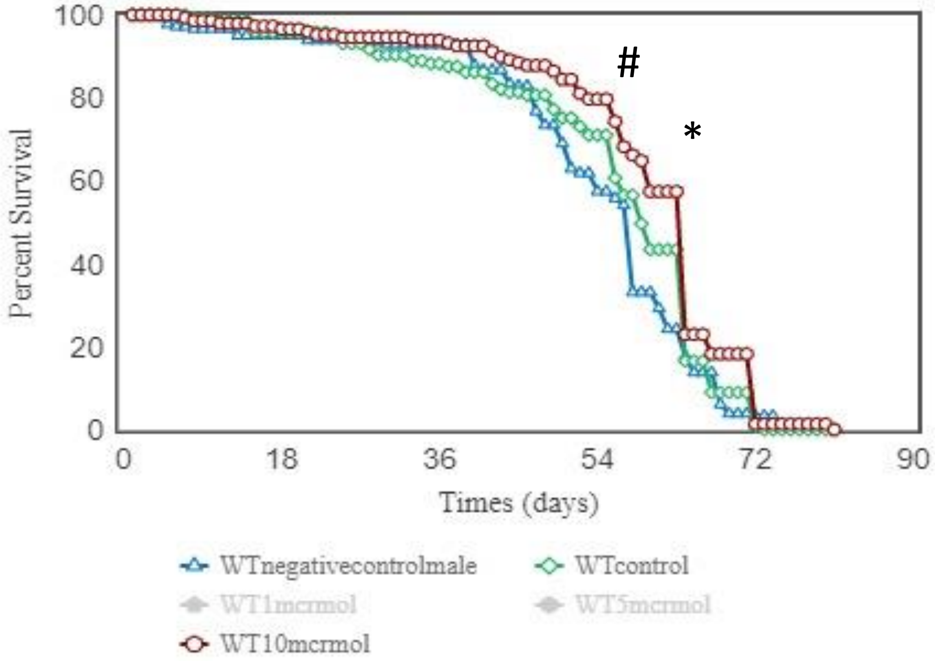

* - p = 0.0166, according to Log-Rank Test with Bonferroni correction (comparison with DMSO control)

# - p = 0.0000094, according to Log-Rank Test with Bonferroni correction (comparison with negative control)

Supplementary figure S4 KS15-treated females' survival

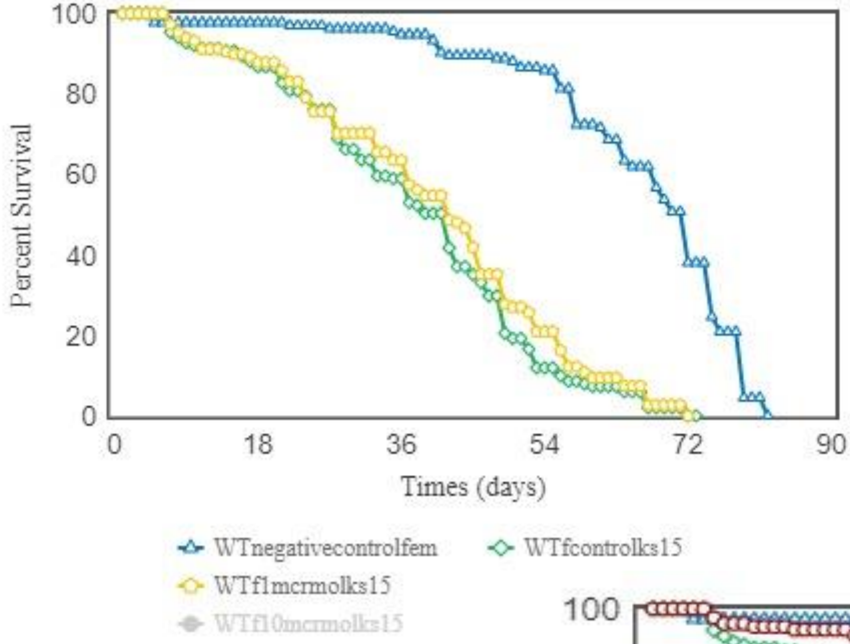

a

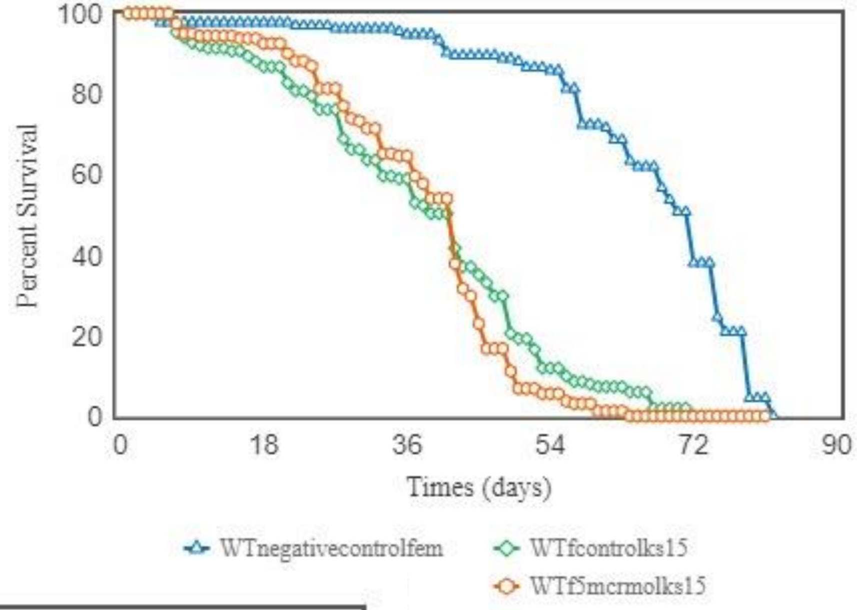

b

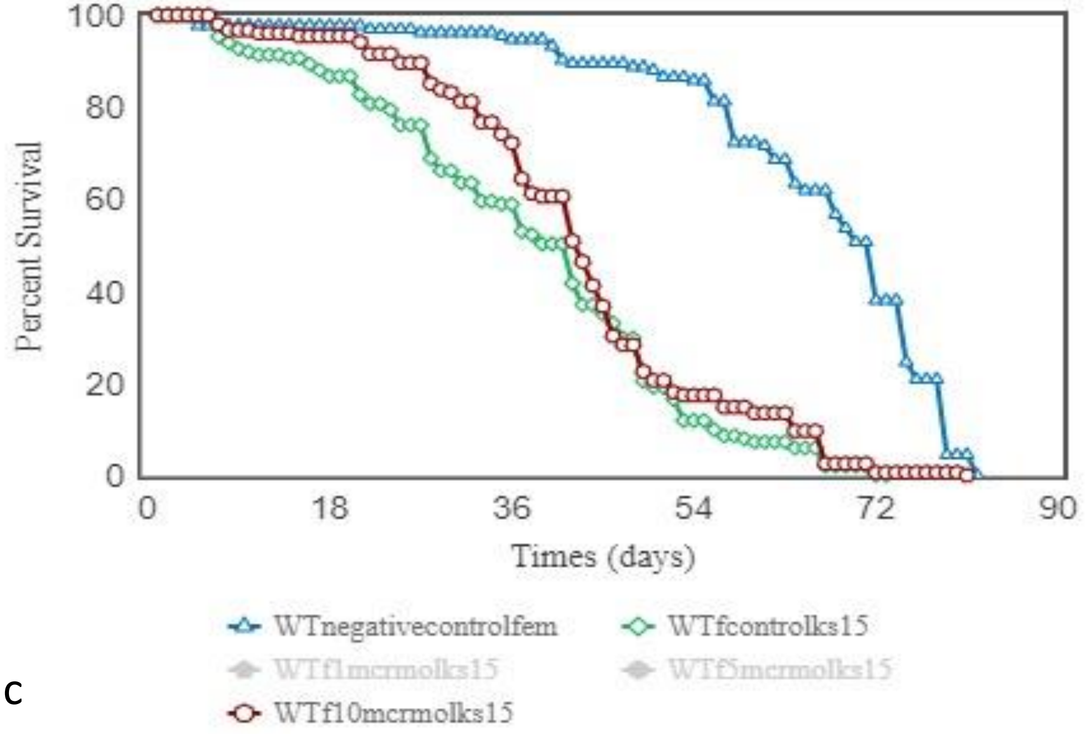

c
